# A new troglobitic species of the genus *Troglocoelotes* Zhao & S. Li, 2019 (Araneae, Agelenidae, Coelotinae) from Guizhou, China

**DOI:** 10.3897/BDJ.11.e103265

**Published:** 2023-07-14

**Authors:** Guchun Zhou, Ru-rui YE, Zhe Zhao

**Affiliations:** 1 School of life Sciences, National Navel Orange Engineering Research Center, Gannan Normal University, Ganzhou, China School of life Sciences, National Navel Orange Engineering Research Center, Gannan Normal University Ganzhou China; 2 Qiandongnan Cave & Rescue Association, Kaili, China Qiandongnan Cave & Rescue Association Kaili China; 3 Institute of Zoology, Chinese Academy of Sciences, Beijing, China Institute of Zoology, Chinese Academy of Sciences Beijing China

**Keywords:** Asia, eyeless, funnel-web spider, taxonomy, troglomorphism

## Abstract

**Background:**

*Troglocoelotes* Zhao & S. Li, 2019 is the only known genus of Coelotinae of which all species have deep morphological adaptations to the subterranean environment, such as depigmentation of body, degenerated or absent eyes and, frequently, with attenuated bodies and/or appendages. Four species of *Troglocoelotes* have been reported from Guizhou Province, China.

**New information:**

A new funnel-web spider of the genus *Troglocoelotes* is described and illustrated on the basis of a single female specimen from Tongren City, Guizhou: *Troglocoelotessinanensis* sp. nov. Additionally, photos of the collection site and a distribution map are also provided.

## Introduction

*Troglocoelotes* Zhao & S. Li, 2019 comprises a total of nine species, that were collected from the deep caves located in the Guizhou Province and Guangxi Zhuang Autonomous Region of China (World Spider Catalog 2023). Specifically, in the Guizhou Province, the following species have been reported: *T.banmenensis* Zhao & S. Li, 2019, *T.proximus* (Chen, Zhu & Kim, 2008), *T.qixianensis* Zhao & S. Li, 2019 and *T.tortus* (Chen, Zhu & Kim, 2008). In the Guangxi Zhuang Autonomous Region, the following five species were recorded: *T.bailongensis* Zhao & S. Li, 2019, *T.liangensis* Zhao & S. Li, 2019, *T.nongchiensis* Zhao & S. Li, 2019, *T.yosiianus* (Nishikawa, 1999) and *T.yumiganensis* Zhao & S. Li, 2019. The majority of the known species are located in subterranean karst formations situated along the boundary of these two regions. All nine species exhibit a degree of ecological segregation, being restricted to distinct cave systems and not found in other subterranean habitats within the same geographic region. *Troglocoelotes* can be diagnosed by chelicerae having three promarginal and two retromarginal teeth, eyes reduced or absent, epigynal teeth located anteriorly or medially, spermatheca usually binary and located retrolaterally (Wang 2002, Zhu et al. 2017, Li et al. 2019a).

**Figure 1.**
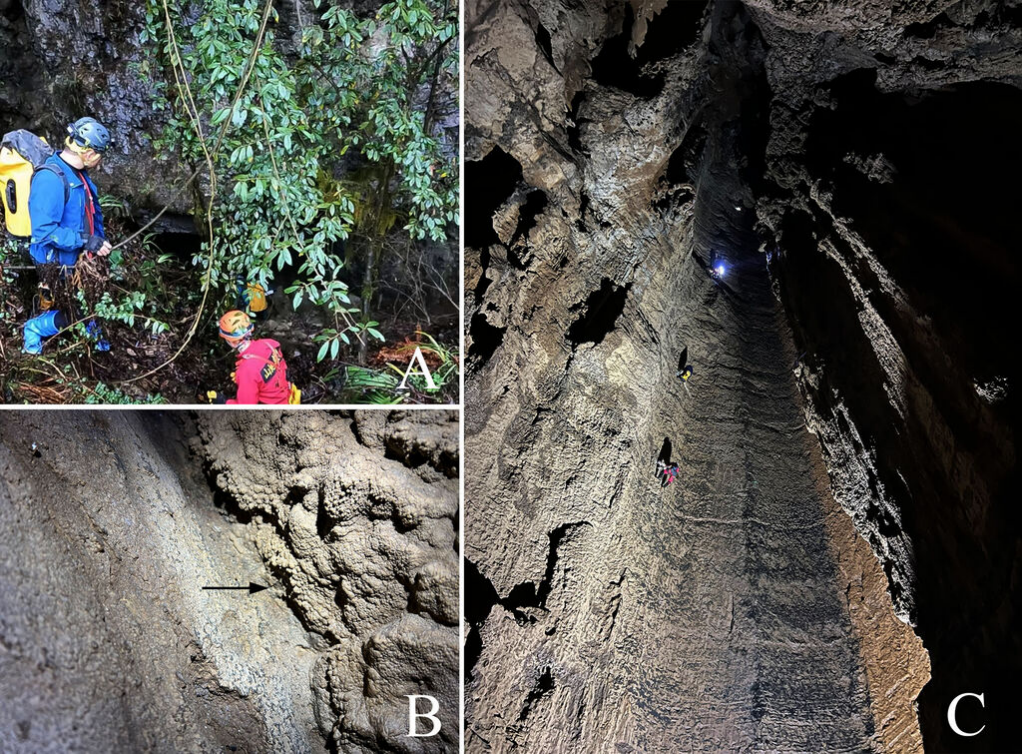
Habitat photos of Huangjin Cave (photographed by Rurui Ye). **A** Entrance of Huangjin Cave; **B** Habitat of the new species; **C** Vertical shaft of Huangjin Cave.

Huangjin Cave is located in Gaofeng Village, Guizhou Province. The cave is about 130 metres long, with a small entrance (Fig. 1A) and a vertical shaft about 100 metres deep (Fig. 1C). After descending through four small cliffs and narrow cracks, a large platform and a pool of water appear at the bottom of the shaft. A new species belonging to *Troglocoelotes* that was collected at the edge of platform (Fig. 1B) at the deepest dark environment of the cave is described in this study.

## Materials and methods

The specimen was collected by handpicking and preserved in 95% ethanol. After dissection, the epigyne was cleared in trypsin enzyme solution before examination and photography. The specimen was examined and measured using an Olympus BX41 stereomicroscope. Photos were taken with a Kuy Nice CCD mounted on an Olympus BX41 and stacked with Helicon Focus software (v.3.10). The map was created using ArcMap 10.2 and then edited using Adobe Photoshop CS2 Extended. Leg measurements are given in the following order: total length (femur, patella + tibia, metatarsus, tarsus). All measurements are given in millimetres (mm). The terminology used in text and figure legends follows Li et al. (2019a). The specimen studied is deposited in the Taxidermy Museum of Gannan Normal University, Ganzhou City, China (GNNU).

## Taxon treatments

### Troglocoelotes sinanensis

Zhou & Zhao, 2023 sp. nov.

C8848107-20A2-5548-BEFF-BD218399AAD3

C1061901-6AE9-4D7E-912E-2A2D8A9621DF

#### Materials

- **Type status:** Holotype. **Occurrence:** catalogNumber: GZTR-230102-1; individualCount: 1; sex: female; lifeStage: adult; occurrenceID: 121C5F31-FAE2-5F04-AF69-9DE147BA39B4; **Taxon:** kingdom: Animalia; phylum: Arthropoda; class: Arachnida; order: Araneae; family: Agelenidae; genus: Troglocoelotes; **Location:** continent: Asia; country: China; countryCode: CN; stateProvince: Guizhou; county: Sinan; locality: Sunjiaba Town, Gaofeng Village, Huangjin Cave; verbatimElevation: 695m; verbatimLatitude: 27.9113°N; verbatimLongitude: 108.2895°E; **Identification:** identifiedBy: Guchou Zhou; **Event:** samplingProtocol: by hand; year: 2023; month: 1; day: 2; habitat: cave; eventRemarks: cave darkness, 17℃, RH 93%; **Record Level:** institutionID: Taxidermy Museum of Gannan Normal University; institutionCode: GNNU; collectionCode: Rurui Ye

#### Description

**Figure 2.**
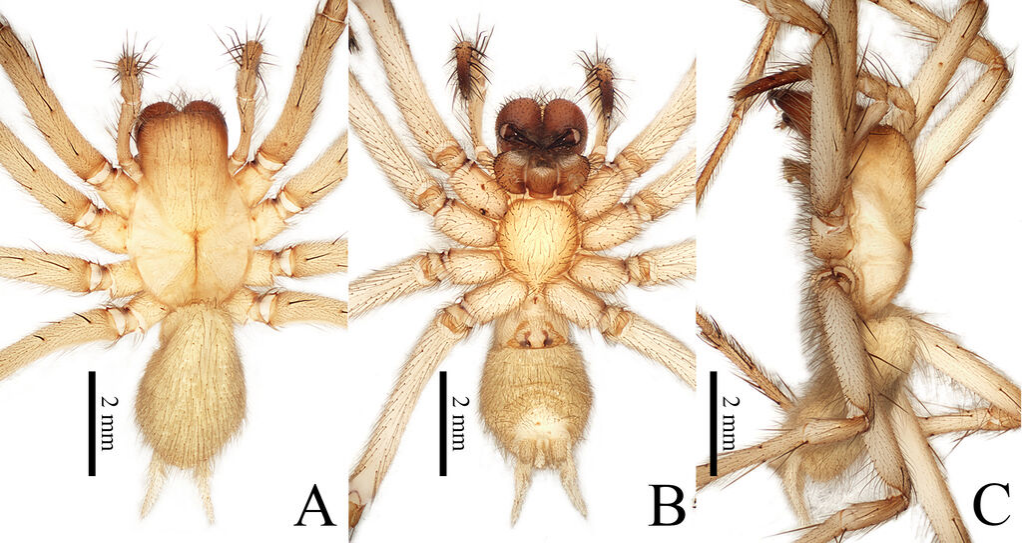
*Troglocoelotessinanensis* sp. nov., the habitus of holotype female. **A** Dorsal view; **B** Ventral view; **C** Lateral view. Scale bars: 2 mm (A–C).

**Figure 3.**
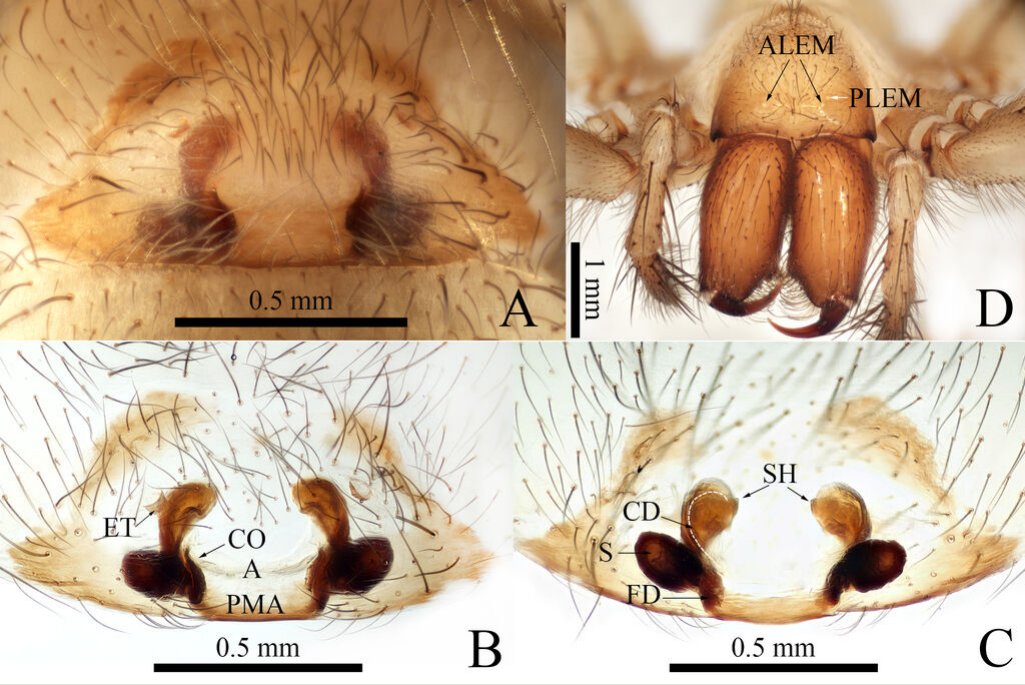
*Troglocoelotessinanensis* sp. nov., holotype female. **A** Epigyne, ventral view; **B** Same, ventral view; **C** Same, dorsal view; **D** Cephalothorax, frontal view. Abbreviations: A = atrium; ALEM = pale marks of anterior lateral eye; CD = copulatory duct; CO = copulatory opening; ET = epigynal tooth; FD = fertilisation duct; PLEM = pale marks of posterior lateral eye; PMA = posterior margin of atrium; S = spermatheca; SH = spermathecal head. Scale bars: 0.50 mm (A–C), 1 mm (D).

**Female** (**holotype**): Total length 6.92. Carapace 3.73 long, 2.38 wide. Cephalic region pale yellowish-brown with brown margin, thoracic region ivory. Cervical and radial furrows distinct and longitudinal fovea very long and aciculate. Abdomen 3.19 long, 2.15 wide; yellowish and covered with numerous fine hairs. Eyes strongly degenerated, reduced to white spots (Fig. 3D). Clypeus height 0.31. Chelicerae basally light brown and inflated. Chelicerae teeth black. Chelicerae each with three promarginal teeth and two retromarginal teeth. Maxillae and labium brown. Sternum 1.86 long, 1.59 wide, covered with hairs of varying lengths. Leg yellowish without annular striations; femur with several fine hairs (Fig. 2A), legs ventrally with numerous fine hairs (Fig. 2B). Palp and legs measurements: palp 5.03 (2.34, 1.31, 1.38); leg I: 13.92 (3.42, 4.89, 3.38, 2.23); leg II: 12.88 (3.38, 4.50, 2.85, 2.15); leg III: 11.35 (2.88, 4.16, 2.96, 1.35); leg IV: 16.55 (3.73, 5.05, 5.31, 2.46).

**Epigyne**: Epigynal teeth fingernail-shaped (Fig. 3A, B); copulatory distinct (Fig. 3B); copulatory duct slender, curved before reaching ellipsoidal spermatheca (Fig. 3C); spermatheca ellipsoidal with a sprout shape (Fig. 3C); spermathecal head small (Fig. 3C); fertilisation duct small and indistinct (Fig. 3C).

**Male**: Unknown.

#### Diagnosis

The female of *Troglocoelotessinanensis* sp. nov. resembles *T.proximus* (Chen, Zhu & Kim, 2008), but can be distinguished by the morphology of the spermathecae: 1) spermatheca ellipsoidal with a sprout shape in *T.sinanensis* sp. nov. (Fig. 3C), vs. in a discoidal shape in *T.proximus* (Chen et al. (2008), fig. 8); 2) upper half of spermathecae away from each other in *T.sinanensis* sp. nov. (Fig. 3C), vs. close together in *T.proximus* (Chen et al. (2008), fig. 9).

#### Etymology

The specific name refers to the type locality, adjective.

#### Distribution

**Figure 4.**
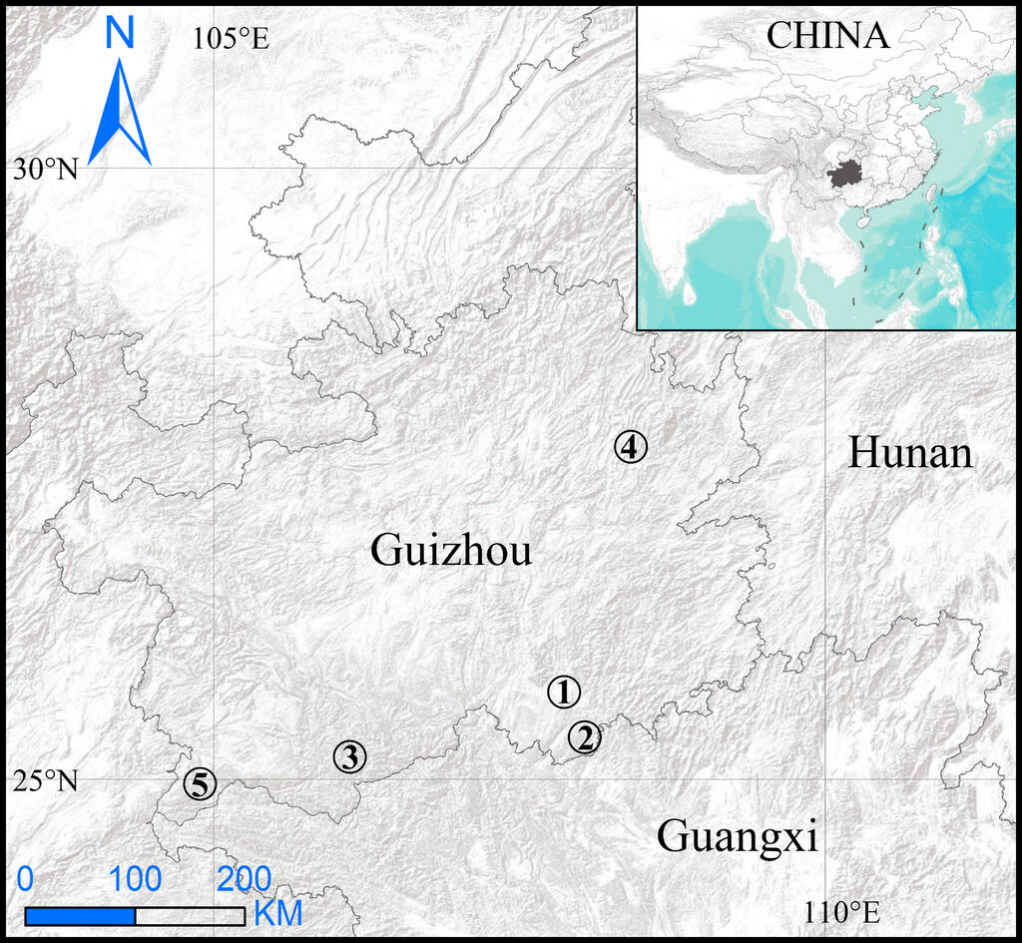
Distribution records of *Troglocoelotes* species from Guizhou Province, China. 1. *T.banmenensis* Zhao & Li, 2019; 2. *T.proximus* (Chen, Zhu & Kim, 2008); 3. *T.qixianensis* Zhao & Li, 2019; 4. *T.sinanensis* sp. nov.; 5. *T.tortus* (Chen, Zhu & Kim, 2008).

Known only from the type locality in Guizhou, China (Fig. 4).

#### Taxon discussion

Huangjin Cave is a vertical cave that is very difficult for humans to explore, so the discovery of this new species was accidental. This species is the deepest subterranean species found in the genus to date. The degenerated eyes and the pale body of *Troglocoelotessinanensis* sp. nov. shows that it is a species well-specialised to subterranean life. Cave adaptation not only causes troglomorphism (morphological adaptation), but also promotes reproductive isolation and speciation (Cizauskas et al. 2022). Considering the karst topography of eastern Guizhou, many new species of *Troglocoelotes* are likely to be found in this area in the future.

## Discussion

Many species of Coelotinae are related to dark cave habitats, but the majority are found at the entrance or shallow subsurface (Zhao and Li 2016, Li et al. 2018, Li et al. 2019b). The description of the newly-discovered species suggests that the genus *Troglocoelotes* has a broad distribution in the southern regions of China, but its diversity remains largely unexplored due to the adaptation of this group to subterranean habitats. Since no epigean species of this genus have been found yet, it may be difficult to use a dispersal model to explain the distribution of *Troglocoelotes* species. Understanding the origins of distant, but closely related subterranean species remains a challenge, particularly amongst monophyletic subterranean species (Mammola et al. 2020). According to zoogeographic research, the regional distribution of coelotin spiders at the genus level is linked to the paleogeological and paleoclimatic changes in Eurasia (Zhao and Li 2017, Zhao et al. 2022). The divergence and speciation of the troglobitic species in question may be associated with karstification in the south-eastern region of China (Zhao et al. 2022).

## Supplementary Material

XML Treatment for Troglocoelotes sinanensis
